# The potential role of indomethacin in COVID-19 management: A systematic review of therapeutic and mechanistic evidence

**DOI:** 10.1016/j.nmni.2026.101771

**Published:** 2026-05-22

**Authors:** Mokhtar Yaghobi, Milad Jalilian

**Affiliations:** Department of Nursing, Faculty of Nursing and Midwifery, Kurdistan University of Medical Sciences, Sanandaj, Iran

**Keywords:** Indomethacin, COVID-19, SARS-CoV 2, NSAIDs, Prevention, Treatment

## Abstract

**Background:**

Indomethacin has been proposed as a repurposed agent for COVID-19 due to its anti-inflammatory and potential antiviral effects against coronaviruses. However, the available evidence is limited, heterogeneous, and largely indirect, with a large observational NSAID study dominating the sample size without evaluating indomethacin-specific efficacy.

**Methods:**

We conducted a PRISMA 2020–compliant systematic review registered in PROSPERO (CRD420251272994). Major databases and trial registries were searched from January 2019 to July 2025 using reproducible strategies. Eligible studies included randomized trials, observational studies, case series, and experimental investigations. Clinical and mechanistic evidence were synthesized separately, and risk of bias was assessed using RoB 2 and ROBINS-I tools.

**Results:**

Eight studies were included: two randomized trials, three observational studies, one case series, one in vitro study, and one pharmacokinetic/pharmacodynamic modeling study. The largest dataset (n = 536,423) provided indirect safety data only. Small clinical studies suggested faster symptom resolution and shorter hospitalization in mild-to-moderate cases, but findings were inconsistent and at risk of bias. Experimental studies supported antiviral mechanisms, including inhibition of viral RNA synthesis and host translation pathways. No study evaluated prophylactic use.

**Conclusion:**

Evidence for indomethacin in COVID-19 remains limited and low certainty. Safety conclusions should be cautious given incomplete reporting and known NSAID risks. Larger, well-designed randomized trials are required.

## Highlights

• Clinical studies suggest reduced symptoms and shorter hospitalization.
• No clinical evidence supports indomethacin for COVID-19 prevention.
• Experimental data show antiviral and immunomodulatory mechanisms.
• Combination therapy with ketotifen enhanced antiviral activity against SARS-CoV-2.
• Indomethacin–ketotifen combination warrants further clinical study.

## 1 Introduction

Coronavirus disease 2019 (COVID-19), caused by SARS-CoV-2, is a multisystem disorder primarily affecting the respiratory system but also involving gastrointestinal, cardiovascular, and neurological functions [[1], [2], [3], [4], [5], [6]]. In severe cases, an exaggerated immune response and cytokine storm play a central role in disease progression and mortality [7,8]. Patients may present with a wide range of symptoms, including fever, fatigue, anosmia, respiratory distress, and coagulation abnormalities [2,[9], [10], [11]]. Despite advances in vaccination, the pandemic highlighted the urgent need for effective and accessible pharmacological interventions.

Drug repurposing has emerged as a pragmatic strategy, particularly focusing on agents with established safety profiles and widespread availability [[12], [13], [14]]. Among these, non-steroidal anti-inflammatory drugs (NSAIDs) have attracted attention due to their anti-inflammatory properties. Indomethacin, a well-known non-selective cyclooxygenase (COX-1 and COX-2) inhibitor, has been widely used since the 1960s for its analgesic, antipyretic, and anti-inflammatory effects [[15], [16], [17], [18], [19]]. In addition to these conventional actions, indomethacin has demonstrated broad-spectrum antiviral activity against several DNA and RNA viruses, including coronaviruses such as SARS-CoV-1 and canine coronavirus [19].

The potential role of indomethacin in COVID-19 is supported by its proposed dual mechanism: inhibition of viral replication and modulation of the host inflammatory response. Experimental evidence suggests that these antiviral effects may occur through cyclooxygenase-independent pathways, including suppression of viral RNA synthesis and interference with host cellular processes. However, the available clinical evidence remains limited and heterogeneous, with some studies not specifically designed to evaluate indomethacin efficacy.

Therefore, this systematic review aims to critically synthesize clinical and mechanistic evidence on indomethacin in COVID-19, with emphasis on evidence quality, methodological transparency, and the distinction between direct and indirect findings.

## 2 Methods

### 2.1 Study design and protocol registration

This study was conducted as a systematic review in accordance with the Preferred Reporting Items for Systematic Reviews and Meta-Analyses (PRISMA) 2020 statement [20]. The review protocol was prospectively registered in the International Prospective Register of Systematic Reviews (PROSPERO) under the registration number CRD420251272994. The protocol was used to define the review question, eligibility criteria, and planned analytic approach before screening began.

### 2.2 Eligibly criteria

Eligibility was defined using the PICOS framework. We included human studies of any age with confirmed SARS-CoV-2 infection that evaluated indomethacin at any dose, formulation, route, or duration, alone or in combination with other therapies. Randomized controlled trials, quasi-randomized trials, cohort studies, and case-control studies were eligible as clinical evidence. In vitro, animal, and modeling studies were included only when they addressed indomethacin activity against SARS-CoV-2 or closely related coronaviruses and were analyzed separately from clinical outcomes. Studies that evaluated multiple NSAIDs without isolating indomethacin-specific effects were retained only for indirect safety context, not for claims of indomethacin efficacy. Case reports, editorials, narrative reviews, and conference abstracts without original data were excluded.

### 2.3 Information sources and search strategy

PubMed/MEDLINE, Embase, Web of Science, Scopus, ScienceDirect, CENTRAL, Google Scholar, and ClinicalTrials.gov were searched from January 2019 to July 2025 without language restrictions at the search stage. Reference lists of included papers and relevant reviews were also screened. A reproducible core search strategy was applied and adapted to each database: (indomethacin OR INDO OR "indomethacin"[MeSH]) AND (COVID-19 OR SARS-CoV-2 OR coronavirus OR "severe acute respiratory syndrome coronavirus 2″). For mechanistic studies involving related coronaviruses, additional terms included HCoV-229E, HCoV-OC43, SARS coronavirus, CCoV, and MERS-CoV when appropriate.

### 2.4 Study selection

**Fig. 1:**
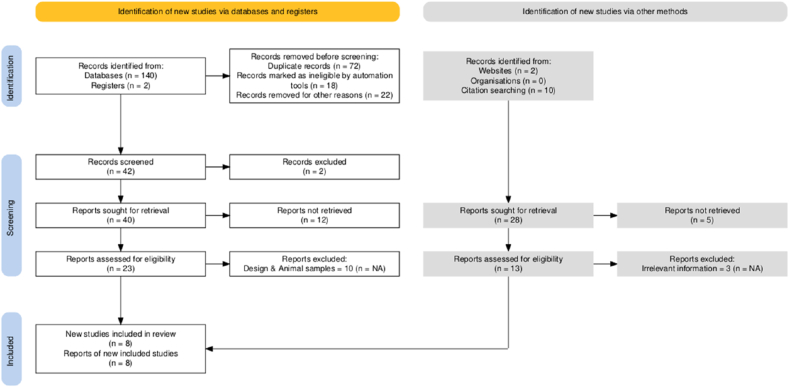
Flow diagram for study selection [39].

All records were imported into EndNote X9 and duplicates were removed. Two reviewers independently screened titles/abstracts and then full texts based on predefined eligibility criteria. Disagreements were resolved by consensus or consultation with a third reviewer. The selection process was summarized using a PRISMA 2020 flow diagram (Fig. 1).

### 2.5 Data collection

Data were extracted independently by two reviewers using a standardized, pilot-tested form. Extracted data were cross-checked, and discrepancies were resolved through discussion.

### 2.6 Data items

Extracted data included study characteristics (author, year, design, country), population details, intervention (dose, duration), comparators, clinical outcomes (e.g., symptom resolution, hospitalization, mortality), and adverse events. For experimental studies, viral and mechanistic outcomes were collected. Clinical and experimental data were analyzed separately.

### 2.7 Risk of bias assessment

Randomized controlled trials were evaluated with RoB 2, and non-randomized comparative studies were evaluated with ROBINS-I [[21], [22], [23]]. For the RCTs, the main concerns were open-label design, limited reporting of allocation concealment, and incomplete blinding procedures. For observational studies, the main concerns were confounding by indication and exposure misclassification, especially in analyses of NSAIDs as a class. Experimental, in vitro, and modeling studies were not assessed with these clinical risk-of-bias tools because such instruments are not applicable to those designs.

### 2.8 Data synthesis

Because the evidence base was small and methodologically heterogeneous, a quantitative meta-analysis was not attempted. Clinical and mechanistic findings were synthesized narratively and interpreted separately so that indirect evidence did not inflate the certainty of indomethacin-specific conclusions.

## 3 Results

### 3.1 Characteristics of included studies

Eight studies were included: two randomized controlled trials, three observational studies, one case series, one in vitro study, and one pharmacokinetic/pharmacodynamic modeling study. The clinical studies were conducted in India, Iran, the United Kingdom, China, and the United States. A key point for interpretation is that the largest sample size came from an NSAID cohort study (Wong et al., n = 536,423), which dominated the total number of participants but did not evaluate indomethacin as an isolated intervention and therefore should be considered indirect safety evidence rather than direct efficacy evidence [24] (Table 1, Table 2, Table 3).

**Table 1:** Characteristics of included studies.

Author (Year)	Country	Study Type	Population/Model	Sample Size	Intervention	Comparator	Main Outcomes	Follow-up
Ravichandran et al. (2022) [25]	India	RCT	Hospitalized mild-to-moderate COVID-19	210	Indomethacin 75 mg (BMI-based dosing)	Paracetamol + SOC	Faster symptom resolution; lower CRP; less oxygen desaturation	14 days
Salmasi et al. (2022) [26]	Iran	RCT	Moderate COVID-19 pneumonia	45	Indomethacin 75 mg SR orally	HCQ + acetaminophen	No significant difference vs comparator	During admission
Wong et al. (2021) [24]	UK	Cohort	NSAID users vs non-users	536,423	NSAIDs including indomethacin	Non-NSAID users	Mortality/severe outcomes; indirect safety evidence	Variable
Bruce et al. (2020) [27]	UK	Cohort	Hospitalized COVID-19 patients	1222	NSAIDs including indomethacin	Non-NSAID users	Mortality; indirect safety evidence	In-hospital
Kanakaraj et al. (2020) [14]	India	Case series	Kidney transplant recipients	12	Low-dose oral indomethacin	None	Short-term symptom improvement; limited reporting	Short-term
Kiani et al. (2021) [28]	USA	In vitro	SARS-CoV-2 cell cultures	NA	Indomethacin ± ketotifen	Control	Reduced viral replication in cell culture	NA
Gomeni et al. (2020) [13]	France	PK/PD modeling	Simulated COVID-19 population	NA	Modeled indomethacin regimens	None	Dose-exposure rationale; simulation only	Simulation
Xu et al. (2020) [29]	China	In vitro/in vivo	SARS-CoV-2/CCoV models	NA	Variable	Control	Antiviral activity in experimental models	NA

**Table 2:** Clinical studies evaluating indomethacin in COVID-19 (human studies only).

Study	Design	Population	Key Findings	Safety Findings
Ravichandran et al. [25]	RCT	Mild–moderate hospitalized COVID-19	Reduced hypoxia, faster symptom relief, ↓ CRP	No serious events reported, but reporting was limited
Salmasi et al. [26]	RCT	Moderate COVID-19 pneumonia	No significant difference vs control	No major events reported
Wong et al. [24]	Cohort	NSAID users	No increased mortality risk	Indirect NSAID-class safety data only
Bruce et al. [27]	Cohort	Hospitalized patients	Neutral mortality effect	Indirect NSAID-class safety data only
Kanakaraj et al. [14]	Case series	Transplant recipients	Shorter hospitalization	Insufficient adverse-event detail

**Table 3:** Risk of bias Assessment.

Study	Tool Used	Overall Risk of Bias	Main concerns
Ravichandran et al. [25]	RoB 2	Some concerns	Open-label design; incomplete blinding/allocation detail
Salmasi et al. [26]	RoB 2	Some concerns	Small sample size; limited methods reporting
Wong et al. [24]	ROBINS-I	Some concerns	Confounding by indication; exposure misclassification
Bruce et al. [27]	ROBINS-I	Some concerns	Observational design; residual confounding
Kanakaraj et al. [14]	ROBINS-I	High	Uncontrolled case series; no comparator

### 3.2 Clinical evidence

The two RCTs directly assessing indomethacin in hospitalized patients with mild-to-moderate COVID-19 suggested possible benefits such as faster symptom relief, lower inflammatory markers, and shorter recovery time in one study, while the other trial found no statistically significant difference in oxygen saturation, intubation, or recovery outcomes compared with control therapy [25,26]. Observational studies and case series reported similar directions of effect in selected populations, but these designs were vulnerable to confounding and selection bias [14,27] (Table 2). None of the included studies directly tested indomethacin for pre-exposure or post-exposure prophylaxis.

### 3.3 Experimental and mechanistic evidence

In vitro and modeling studies supported biological plausibility for indomethacin by showing inhibition of coronavirus replication, suppression of viral RNA synthesis, modulation of host proteins such as PGES-2, and activation of host antiviral pathways including PKR/eIF2α-mediated inhibition of viral protein translation [13,19,[28], [29], [30], [31], [32], [33], [34], [35]]. Recent work also suggested activity against seasonal human coronaviruses at late stages of replication and provided additional support for host-mediated antiviral effects [32]. These findings are mechanistically interesting but cannot be interpreted as clinical efficacy evidence.

### 3.4 Risk of bias and methodological quality

The RCTs were judged to have some concerns because of open-label design and incomplete reporting of blinding and allocation procedures. Observational studies were affected by confounding by indication and the difficulty of isolating indomethacin-specific effects from broader NSAID exposure. The case series was at high risk of bias because it lacked a comparator group and included only a small, highly selected population. Adverse-event reporting was inconsistent across studies, limiting confidence in safety conclusions. Table 3.

## 4 Discussion

This review was designed to address a persistent problem in the indomethacin literature: mechanistic enthusiasm has often outpaced clinically actionable evidence. The main contribution of the present synthesis is therefore not a claim of proven efficacy, but a clearer separation of direct clinical evidence from indirect observational safety data and experimental plausibility.

The clinical signal for benefit was modest and inconsistent. The two RCTs suggested that indomethacin may improve selected outcomes in hospitalized patients with mild-to-moderate COVID-19, but one study was positive while the other was neutral, and both were limited by small sample sizes and methodological constraints [25,26]. Observational studies were not designed to isolate indomethacin specifically, and the very large OpenSAFELY cohort contributed mostly to safety context rather than efficacy inference [24,27]. For that reason, the total sample size should not be interpreted as evidence that thousands of patients received indomethacin in a rigorous comparative trial.

Mechanistic findings remain the strongest part of the evidence base. Multiple studies have shown that indomethacin can suppress coronavirus replication through cyclooxygenase-independent pathways, including inhibition of viral RNA synthesis, modulation of PGES-2-associated host interactions, and PKR/eIF2α-mediated suppression of viral protein translation [19,28,[30], [31], [32], [33], [34], [35]]. Recent experimental work reporting activity against seasonal human coronaviruses is consistent with this broader host-mediated antiviral profile [32]. These data help explain why indomethacin continues to attract interest, but they do not substitute for clinical efficacy data.

The safety profile requires more careful wording than in the original manuscript. No included study consistently reported a strong signal of severe short-term toxicity, but adverse-event ascertainment was incomplete and often underpowered. This is especially important because NSAIDs, including indomethacin, are associated with well-established gastrointestinal, renal, and cardiovascular risks, particularly in older patients, those with dehydration, renal impairment, cardiovascular disease, or concomitant anticoagulant use [[36], [37], [38]]. Accordingly, the absence of reported serious events in the included studies should be interpreted as incomplete evidence rather than proof of safety.

The review also clarifies an important conceptual limitation: no included study directly tested indomethacin as pre- or post-exposure prophylaxis. Any preventive role remains speculative and would need to be established in dedicated trials with infection-related endpoints. This point is especially relevant because some mechanistic discussions in the literature blur the distinction between prevention, early treatment, and symptom mitigation.

### 4.1 Strengths and limitations

This study has several strengths, including prospective registration, transparent eligibility criteria, and separate synthesis of clinical and mechanistic evidence. Its limitations are the small number of direct indomethacin trials, heterogeneity of study designs, the inability to meta-analyze the results, and the risk that indirect NSAID studies can be mistaken for indomethacin-specific evidence. In addition, the literature remains susceptible to publication bias because positive experimental studies are more likely to be reported than negative or null findings.

### 4.2 Implications for future research

Future studies should prioritize adequately powered, placebo-controlled randomized trials with predefined clinical endpoints, standardized dosing, explicit safety monitoring, and transparent reporting of co-interventions. Trials should distinguish therapeutic treatment from prophylaxis, enroll clearly defined patient subgroups, and report adverse events using harmonized criteria. Combination strategies such as indomethacin plus ketotifen remain hypothesis-generating only and should be tested experimentally before any clinical recommendation is made.

## 5 Conclusion

Indomethacin remains an interesting repurposing candidate for COVID-19 because of its plausible antiviral and anti-inflammatory mechanisms. However, the direct clinical evidence is limited, heterogeneous, and not strong enough to support routine use or broad safety claims. The available studies justify further randomized research, but at present indomethacin should be viewed as a promising hypothesis rather than an established therapy for COVID-19.

## Ethical approval statement

It is a systematic review of previously published literature and did not involve any new research with human participants or animals. The review protocol was prospectively registered in the PROSPERO database (Registration number: CRD420251272994) to ensure methodological transparency and rigor.

## Availability of data and materials

The data from this study are available for rational reason.

## Funding and financial disclosure

This study did not receive any financial support from any organization, or institute or personal financial interests.

## CRediT authorship contribution statement

**Mokhtar Yaghobi:** Conceptualization, Data curation, Formal analysis, Funding acquisition, Investigation, Methodology, Project administration, Resources, Software, Supervision, Validation, Visualization, Writing – original draft, Writing – review & editing. **Milad Jalilian:** Conceptualization, Data curation, Formal analysis, Funding acquisition, Investigation, Methodology, Project administration, Resources, Software, Supervision, Validation, Visualization, Writing – original draft, Writing – review & editing.

## Declaration of competing interest

The authors declare that they have no known competing financial interests or personal relationships that could have appeared to influence the work reported in this paper.
